# Annual Meeting of Massachusetts Veterinary Association

**Published:** 1885-07

**Authors:** L. H. Howard

**Affiliations:** Secretary


### ANNUAL MEETING OF MASSACHUSETTS VETERINARY ASSOCIATION.

The second annual meeting of the Massachusetts Veterinary Association was held at Young's Hotel, Boston, Wednesday evening, April 1st.

The President, Dr. W. Bryden, occupied the chair, and there were present thirteen members, viz.: Drs. Billings, Blackwood, Bunker, Alderman, Harrison, Osgood, Skally, J. S. Saunders, Sherman, Winslow, Winchester and Howard.

After reading of minutes of the previous meeting, and their adoption, the name of A. W. Clement, V.S., was proposed for membership, his credentials having been previously favorably reported on by the executive committee.

On ballot he was unanimously elected to membership.

Election of officers for the ensuing year was next on the order of business and resulted as follows, the vote being a unanimous one: For President, F. S. Billings, V.M.; for Vice-President, J. S. Saunders, D.V.S.; for Secretary and Treasurer, L. H. Howard, D.V.S.; for Executive Committee, J. M. Skally, V.S., W. Bryden, V.S., W. T. Simmons, M.R.C.V.S.

On retiring from the chair Dr. Bryden, in a few remarks, kindly thanked the officers and members for their hearty co-operation in this, the first year's work of our association, and predicted for us a most successful future.

Dr. Billings assumed the chair, and a vote of thanks was tendered the retiring officers.

At the suggestion of the President it was *voted* that a committee be appointed to consider the matter of procuring a charter for the Association.

According to the remarks of some of the gentlemen, the present session of the Legislature is already too-far advanced to grant a *special* charter, that is, by legislative act, though a charter under the *general laws* may be obtained at any time.

The general sentiment seemed to be in favor of a special charter, but after some discussion of the subject it was left to the discretion of the committee appointed, viz.: Drs. Bunker, Bryden and Winchester.

Thus far all meetings of the Associations having been held in Boston, Dr. Billings suggested that an assembly in another part of the State might be productive of good, by creating in other sections an interest in our work and meetings.

Dr. Winchester suggested as a compliment to Dr. Osgood, of Springfield, who has been a very regular attendant at our meetings, and a much-interested member, that the next meeting be held at Springfield.

On Dr. Winchester's motion to that effect, it was *voted* that the next meeting of this Association be held at Springfield: the date of meeting was afterward appointed May 1st, at 8 oclock, P.M., Dr. Osgood being asked to make all necessary arrangements for our accommodation, etc.

The essayist will be Dr. Billings, who will demonstrate Koch's method of bacteria cultivation.

On motion of Dr. Osgood it was *voted* that the members of the medical profession in Springfield, the Connecticut Veterinary Medical Society, Drs. J. & Geo. Penniman, of Worcester, and Dr. Brackin, of Pittsfield, be invited to be present.

Dr. Bunker then exhibited a very interesting pathological specimen, viz., an embolism of the femoral artery, nearly eight inches in length.

The history of this case in brief is, that about six weeks previous the animal, a chestnut gelding, eight years old, showed lameness in right hind leg. He would start from the stable sound and begin to show lameness when he had travelled about a-third of a mile, which lameness kept increasing as he went further, till it became very severe. On being allowed to stop, the animal would raise and lower the leg in an uneasy manner for a few moments, and finally remain quiet; the temperature of this leg being much lower than the other, in fact *cold.* These symptoms continued with more or less variance for six weeks, when the animal was destroyed and the post-mortem examination revealed the embolism mentioned.

At eight o'clock dinner was announced, and the company adjourned and partook of a very bounteous repast, some two hours being spent at table in consumption of the edibles, and listening to very pleasant toasts and after-dinner remarks.

The meeting, again called to order, listened to the reading of a paper by Dr. Harrison, on "Amputation of the Penis."

The essayist noted the indications for operation, calling attention particularly to carcinomatous affections. He then described in detail the *modus operandi*, recommending that the *urethra* be not divided at the point of amputation of the *body* of the penis, but that it be left projecting about an inch, this portion to be divided and the flaps secured back by sutures.

He mentioned four cases in which this operation had been successful and afforded permanent relief; and one case in which the amputation had been effected with the ecraseur, dividing the entire structure of the penis at a given point, the result being fatal.

Some discussion of the subject took place, participated in by Drs. Bryden, Saunders, Winchester, Skally, Osgood and Billings, and it was *voted*, on motion of Dr. Osgood, that the discussion be continued at the next meeting.

A vote of thanks was tendered the essayist, and the meeting was adjourned.

L. H. Howard, Secretary.

Meeting at Springfield, Mass.

The regular monthly meeting was held at the Massasoit House, Springfield, Mass., Friday evening, May 1st.

President F. S. Billing occupied the chair, and there were present Doctors M. Bunker, of Newton; J. E. Gardner, of Greenfield; F. H. Osgood, of Springfield; J. S. Saunders and L. H. Howard, of Boston; J. F. Winchester, of Lawrence, and Charles Winslow, of Rockland; also, as invited guests, P. LeB. Stickney M.D., and W. W. Gardner, M.D., of Springfield; Dr. A. R. Rice, Chairman Springfield Board of Health; Dr. Forrest, of Rockland; Dr. Bland, Secretary Connecticut Veterinary Medical Society; Mr. W. H. Wilkinson, of "Brightside Farm," Holyoke; Messrs. Myrick, of *N. E. Homestead*, Lyman, of *Springfield Republican*, and Geddings, of *Springfield Union.*

The record of previous meeting was read and accepted, and the general order of business was omitted to listen to a paper by Dr. Billings, Part I. being an essay on "State Medicine," and Part II. a demonstration of Koch's method of bacteria cultivation.

All present were very much interested, and at the conclusion of Dr. Billing's remarks a unanimous vote of thanks was tendered him.

Dr. Stickney, of Springfield, said that he had been very much interested both in the paper on state medicine and in the practical illustration of bacteria cultivation, and thought that the address on the former subject ought to be published broadcast.

He also remarked that veterinary medicine ought to be more fully recognized, and that he was ready, as a prantitioner in the department of human medicine, to extend to it the right hand of fellowship, appreciating fully how its investigations and results can assist those in the sister branch of the same great science.

Dr. A. R. Rice, Chairman of the Springfield Board of Health, said that he wished to endorse the remarks of Dr. Stickney and to compliment highly the paper on state medicine.

He said that he was ashamed of the State of Massachusetts not having a law to regulate the practice of medicine, becoming, as it is, the "Botany Bay" for charlatans and quacks.

Dr. W. W. Gardiner, of Springfield, remarked that he had been a member of the Massachusetts Medical Society for many years, but had never yet listened to a paper which he felt was of so much importance as the one he had heard this evening.

Mr. Myrick, of the *Homestead*, spoke of the increasing interest in veterinary matters which he had noticed among the farmers, and said that the increasing demand for veterinary surgeons was a sign of the times.

Dr. Thomas Bland andDr. Forrest complimented highly the papers to which they had listened, and expressed their appreciation of the good fortune enabling them to be present at a meeting of the Massachusetts Veterinary Association.

Dr. Bunker, of Newton, then exhibited a pathological specimen, œdema of the glottis in a cow. The glottis was entirely closed, the cellular tissue all about being filled with serum, larynx somewhat inflamed.

The symptoms were sudden in their development, respiration becoming rapidly hurried and short, tracheotomy not being performed becanse the animal was destroyed for the butcher at the end of twelve hours.

At ten o'clock the company adjourned to the banquet-hall, where the next two hours were spent at dinner, after which we listened to very pleasant after-dinner speeches by the different members and their guests.

The meeting was subsequently called to order, and quite a discussion took place in regard to the "subscription plan" as pursued by veterinary institutions in general, and Harvard Veterinary School in particular.

It seemed to be the general opinion that the system was decidedly unjust to the practitioner, and derogatory and detrimental to the best interests of the profession.

On motion of Dr. Howard, it was unanimously *voted* that a committee of three be appointed by the chair, to prepare resolutions censuring the system.

The chair appointed as that committee: Doctors Saunders, Bunker and Howard.

A unanimous vote of thanks was extended to Dr. Osgood for his courteous attention to the Association while in his city.

The chair appointed as the essayist for the next meeting Dr. Alderman, and Dr. Bryden for the next meeting following.

No other business coming before the meeting, it was adjourned, all present expressing the opinion that the meeting had been an enjoyable one, and fraught with much good to our Association. L. H. Howard, Secretary.

